# The effect of perceived discrimination on the health of immigrant workers in Spain

**DOI:** 10.1186/1471-2458-11-652

**Published:** 2011-08-17

**Authors:** Andrés A Agudelo-Suárez, Elena Ronda-Pérez, Diana Gil-González, Carmen Vives-Cases, Ana M García, Carlos Ruiz-Frutos, Emily Felt, Fernando G Benavides

**Affiliations:** 1 Faculty of Dentistry, University of Antioquia, Calle 64 N° 52-59. Medellin, Antioquia, Colombia; 2 Preventive Medicine and Public Health Area, University of Alicante, Campus de San Vicente del Raspeig s/n, Alicante, 03690, Spain; 3 Centre for Research in Occupational Health, Parc de Recerca Biomèdica de Barcelona (PRBB), C/Dr. Aiguader 88, Barcelona 08003, Spain; 4 CIBER Epidemiology and Public Health, Spain; 5 Observatory of Health Policies and Health, University of Alicante, Campus de San Vicente del Raspeig s/n, Alicante, 03690, Spain; 6 Department of Preventive Medicine and Public Health, University of Valencia. Av. Tarongers s/n, Valencia, 46022, Spain; 7 Trade Union Institute for Work, Environment and Health (ISTAS), C/Ramon Gordillo 7-1, Valencia 46010, Spain; 8 Department of Environmental Biology and Public Health, University of Huelva. Avenida de las Fuerzas Armadas, S/N. Huelva, 21071, Spain

## Abstract

**Background:**

Discrimination is an important determinant of health inequalities, and immigrants may be more vulnerable to certain types of discrimination than the native-born. This study analyses the relationship between immigrants' perceived discrimination and various self-reported health indicators.

**Methods:**

A cross-sectional survey was conducted (2008) amongst a non-random sample of 2434 immigrants from Ecuador, Morocco, Romania and Colombia in four Spanish cities: Barcelona, Huelva, Madrid and Valencia. A factorial analysis of variables revealed three dimensions of perceived discrimination (due to immigrant status, due to physical appearance, and workplace-related). The association of these dimensions with self-rated health, mental health (GHQ-12), change in self-rated health between origin and host country, and other self-reported health outcomes was analysed. Logistic regression was used adjusting for potential confounders (aOR-95%CI). Subjects with worsening self-reported health status potentially attributable to perceived discrimination was estimated (population attributable proportion, PAP %).

**Results:**

73.3% of men and 69.3% of women immigrants reported discrimination due to immigrant status. Moroccans showed the highest prevalence of perceived discrimination. Immigrants reporting discrimination were at significantly higher risk of reporting health problems than those not reporting discrimination. Workplace-related discrimination was associated with poor mental health (aOR 2.97 95%CI 2.45-3.60), and the worsening of self-rated health (aOR 2.20 95%CI 1.73- 2.80). 40% (95% CI 24-53) PAP of those reporting worse self-rated health could be attributable to discrimination due to immigrant status.

**Conclusions:**

Discrimination may constitute a risk factor for health in immigrant workers in Spain and could explain some health inequalities among immigrant populations in Spanish society.

## Background

Discrimination is considered a determinant of health and health inequalities [1-4]. Discrimination can be defined as one or more members of a socially established group being treated differently (pejoratively) because of his/her/their belonging to that group [5]. Discrimination may be exercised by an individual, a group of individuals, or by public and private organisations when they fail to attend equally to the needs of groups in less favourable socioeconomic situations [5].

From a social epidemiology perspective, it is relevant to analyse how discrimination is reproduced along gender lines, through social class or through ethnicity in order to reach an effective understanding of the phenomenon [6]. The immigrant population, which often represents ethnic groups different from those of the native population, is especially vulnerable to discrimination [7]. Immigrant populations face significant barriers in overcoming social and economic inequalities, in part due to institutional racism and other forms of discrimination, resulting in poor health-related indicators [8].

Scientific research has reported that the lack of a job contract, lack of social support, difficulties in communication, low level of education and cultural identity (cultural mores and values) are factors that may contribute to the discrimination experienced by the immigrant population [2-4,6,9,10]. In addition, scientific evidence associates experiences of discrimination with worse self-perceived health [11], a higher prevalence of chronic diseases [12,13] and mental health problems [14-17]. In the past two decades, Spain has experienced a dramatic influx of immigrants from other countries. Most of them emigrated primarily for economic and work-related reasons [18,19]. The demand for non-skilled labour during this time period has meant that immigrant workers, rather than natives, generally occupy the most precarious and temporary jobs, and their access to more qualified positions has been limited [20-23]. The lack of job mobility, combined with difficulties in financing basic needs and in access to public resources, constitutes a factor of discrimination borne by the immigrant population [9,24].

Nevertheless, the extent to which working conditions and job sector have an effect on the relationship between perceived discrimination and state of health has been scarcely explored [2-4,10]. In a qualitative study consisting of 84 interviews and 12 focus groups with members of immigrant communities in Spain (Romanians, Moroccans, Ecuadorians, Colombians and Sub-Saharan Africans), we found that discrimination from bosses and other employees, as well as discrimination experienced in daily surroundings, affects the job security, physical health, and mental health of immigrants [7]. It is also important to establish the prevalence of discrimination in immigrants with working experience in Spain and the specific association between the types of discrimination experienced and the health status of immigrant workers. Accordingly, in this study we analyse the relationship between several categories of immigrants' perceived discrimination and various self- reported health indicators in a large sample of foreign-born workers in Spain.

## Methods

### Design, data collection and setting

This cross-sectional analysis is a part of the larger ITSAL Project (Inmigración, Trabajo y Salud, the Spanish acronym). A 74 item questionnaire was developed with the aim of gathering information on socio-demographic characteristics, migration processes, employment and working conditions, and physical and mental health of immigrants working in Spain (available upon request). The questionnaire was developed based on the results of a previous qualitative study of the ITSAL Project [20,22] and piloted with a sample of 35 foreign-born workers in order to improve intelligibility and to assess time to completion and internal consistency [25].

The sample of foreign-born workers (n = 2434) consists of individuals from the countries that send the bulk of immigrants to Spain (Morocco, Ecuador, Romania and Colombia). The survey was carried out in four Spanish cities representing major places of immigrants' residence (Barcelona, Huelva, Madrid and Valencia) [26]. Individuals included in the sample were required to meet the following inclusion criteria: residence in Spain for at least one year, active employment in the country for at least three months -excluding some occupations: athletes, artists, students, business executives- and adequate Spanish language abilities sufficient to participate in the interview. Foreign-born workers with Spanish citizenship or those married to a native Spaniard were excluded. Quota sampling methodology [27] was used for each sample, with a quota set by nationality, gender, and area of residence in Spain. This strategy was used in order to obtain statistical data for each group of immigrants. All selected individuals meeting the inclusion criteria were invited to participate in the study and were provided an informational letter explaining their rights and guaranteeing individual confidentiality. Participation was voluntary, with consent implied by the decision to complete the survey.

Face to face interviews were conducted from April to June 2008, with a 55.8% response rate. Surveys, which lasted an average of 30 minutes, were conducted by trained interviewers who made contact with immigrant workers through organisations that work with immigrants, as well as through posters and direct recruitment in a variety of locations such as local stores, metro and bus stations, telephone centres, and markets in immigrants' neighbourhoods. Interviewers received training with the questionnaire and survey techniques prior to the fieldwork in order to facilitate their interactions with immigrant populations [25].

The study protocol was approved by the Ethical Committees of the participating institutions (University Pompeu Fabra of Barcelona, University of Valencia, University of Huelva, University of Alicante and Trade Union Institute for Work, Environmental and Health of Madrid).

### Variable Definitions

For the purposes of this study, perceived discrimination was determined by answers to the question: "Have you ever felt discriminated against?" (Yes/No) with 12 non-exclusive alternatives of response: when looking for a job, for being an immigrant, because of nationality, on the street (or in public spaces), for being undocumented, by the boss, by workmates, by public and private institutions, at the workplace, due to modes of dress, because of sex/gender, and on the basis of physical appearance or skin colour.

To evaluate health status, certain physical and mental health indicators were used separately: 1) self-rated health (How would you rate your current health status?) was categorized as good (good/very good) or poor (fair/poor/very poor); 2) mental health (as assessed by the 12-item General Health Questionnaire; responses scoring > = 3 were classified as poor mental health) [28,29]; and responses to the question: Have you ever had some of these problems: 3) musculoskeletal symptoms: muscle and/or joint pain, tingling, loss of strength and decreased sensitivity (yes/no); 4) headache (yes/no); 5) stress (yes/no); 6) insomnia (yes/no); and 7) anxiety (yes/no). All health questions referred to the year prior to the survey. Information on self-perceived health status in the immigrant's origin country was collected in the same way as self-rated health. A new variable was created (change in self-rated health) according to responses concerning self-rated health in Spain and self-rated health in the origin country (no change-improved/worse). We assigned different scores to each variable (0 good; 1 poor). If the measure of the difference was 0, it was assumed that there was no change, and if the difference was -1, it was assumed that the situation was worse in the host country, and finally if the result was +1, it was assumed that the health situation was better in Spain.

Other variables were included in the analysis as possible confounders: age (< = 24, 25-34, 35-44, 45-54 and > = 55), occupation (manual: those working in service industries, agriculture, or construction; non-manual: professional and the like, government and business managers, administrators, and sales persons), education level (1) without studies/primary or elementary school; 2) secondary: high school, and 3) university and post graduate studies), economic activities (agriculture, industry, construction, services), origin country (Ecuador, Morocco, Romania, and Colombia), length of time in Spain (< 2, 2-6, > 6 years), and legal status for residence and working in Spain (undocumented/documented). Self-perceived health status prior to arriving to Spain (good/poor) was also considered as a potential confounder.

### Data analysis

We regrouped discrimination items into several categories by means of a factor analysis [30]. After testing 1, 2, 3, and 4-factor solutions, the research team decided to use a 3-factor solution, considering the types of discrimination established in the literature of the topic (table 1). Initially, the variables related to discrimination (the 12 non-exclusive alternatives of response) were checked to ensure a statistically significant correlation by means of matrices and associated p values, and we found that all variables were correlated (p < 0.0001). The Bartlett's sphericity test was used to confirm the study's dependent variable, with a value of 0.932 and a p value < 0.0001; this confirmed that the factor analysis as a method is appropriate. Finally, the extraction method used was through principle component analysis using varimax rotation. Once the correspondences in the answers to questions about discrimination had been observed and the scores were obtained in the final matrix (Table 1), the answers were categorised into three types of discrimination: 1) due to immigrant status (items 1 through 5), 2) due to physical appearance (items 10 through 12) and 3) relating to the workplace (items 6 through 9). These categories were not mutually exclusive (One person can perceive more than one type of discrimination).

**Table 1:** Results of the factorial analysis (rotated component matrix) derived from the response alternatives to the questions about perceived discrimination

			Components: self-perceived discrimination		
			
Events/Situations of discrimination^c^	n	%	By immigrant condition	For physical appearance	workplace related
Have you ever felt discriminated?					
Looking for a job	1772	48.2	**0.746**	0.295	0.014
For being and immigrant	1221	50.2	**0.722**	0.084	0.338
Because of nationality	1044	42.9	**0.718**	0.145	0.262
For being undocumented	1064	43.7	**0.679**	0.166	0.154
On the street (public spaces)	1119	46.0	**0.662**	0.289	0.070
By the boss	493	20.3	0.182	0.150	**0.756**
By the workmates	458	18.8	0.104	0.228	**0.709**
At the workplace	772	31.7	0.433	0.151	**0.605**
For public and private institutions	670	27.5	0.361	0.089	**0.538**
For modes of dress	394	16.2	0.200	**0.795**	0.148
For physical appearance, skin colour	635	26.1	0.402	**0.663**	0.103
Because of the sex	259	10.6	-0.002	**0.627**	0.410

(n = 2434)^a,b^

^a ^Extraction method: Principle component analysis. Rotation method: Varimax normalization.

^b ^24.6% of the participants reporting no discrimination and 75.4% reporting at least 1 discrimination event (or one type of discrimination).

^c ^According to the literature and considering the results, the research team assumed the item "looking for a job" as a factor prior to the job experience in Spain and more related with the factor 1 (immigrant condition). In the case of the item: "For public and private institutions", it was grouped with the workplace related discrimination items because in our previous work we have noted that public and private institutions such as trade unions, organizations that work to defend workers' rights, and government institutions that manage the legal situation of immigrant workers often have a direct impact on the workplace conditions of immigrants. (Factor 3).

We used logistical regression to measure the relationship between the three defined types of perceived discrimination and each of the health outcomes of interest, first in a crude analysis and then adjusted to account for possible confounders according to previous literature [2-4,10]. For these analyses, we used a model which includes all the confounders mentioned, and we show the complete adjusted models. Results were recorded as odds ratios (OR) with 95% confidence intervals (95% CI). Finally, the estimation of possible cases of change (worsening) in self-perceived health status attributable to the three defined types of perceived discrimination mentioned above was made by the population attributable proportion (PAP), expressed as a percentage, by the following expression [31]:

$$PAP=\left(\frac{Pe\left(OR-1\right)}{Pe\left(OR-1\right)+1}\right)\times 100$$

Where Pe represents the prevalence of people perceiving each discrimination type and the OR are those obtained from logistic models. Confidence limits for the PAP intervals to 95% were made using a substitution method [32]. All calculations were computed using SPSS 17.0.

## Results

Tables 2 and 3 show the distribution of the three perceived discrimination groups defined in the sample (n = 2434) according to socio-demographic characteristics and health outcomes. A total of 57% of the participants were male, and 65% were younger than 44 years old. The majority had documented immigration status and worked in manual occupations in the construction and service economic sectors. A total of 51.2% of the interviewees had completed secondary levels of education. The majority of those interviewed had been in Spain for 2 to 6 years. A total of 94% of subjects reported their health status as good in their country of origin. 75.4% of participants reported at least one type of discrimination. The most frequently reported category of perceived discrimination was due to immigrant status (72%). Moroccans reported discrimination of all three types more frequently than immigrants from other countries. The most frequent health problems among participants were headache and stress, and the highest frequencies are observed among those who report discrimination related to their condition as immigrants.

**Table 2:** Distribution of the sample of immigrant workers in Spain and prevalence of the three categories of perceived discrimination for socio-demographic characteristics

			Perceived discrimination^a^					
	
	Sample		Due to immigrant status		Due to physical appearance		Workplace related	
	
Socio-demographic characteristics	n	%	n	Prevalence	n	Prevalence	n	Prevalence
Sex								
Male	1395	57.3	1022	73.3	488	35.0	700	50.2
Female	1039	42.7	720	69.3	325	31.3	480	46.2
Age (y)^b^								
< = 24	422	13.3	313	74.2	143	33.9	216	51.2
25-34	1097	45.1	789	71.9	366	33.4	535	48.8
35-44	638	26.2	464	72.7	225	35.3	308	48.3
45-54	222	9.1	145	65.3	63	28.4	98	44.1
> = 55	42	1.7	21	50.0	10	23.8	15	35.7
Legal Status (work and residence)								
Documented	1893	77.8	1324	69.8	638	33.7	894	47.2
Undocumented	541	22.2	421	77.8	175	32.3	286	52.9
Occupation								
Manual	1539	63.3	1111	72.2	536	34.8	770	50.0
Non manual	894	36.7	630	70.5	277	31.0	410	45.9
Educative level^b^								
Non studies/primary studies	770	31.6	576	74.8	308	40.0	412	53.5
Secondary	1247	51.2	883	70.8	392	31.4	593	47.6
University	413	17.0	280	67.8	112	27.1	173	41.9
Economic Activities (Main sectors)^b^								
Agriculture	266	10.9	129	71.1	89	33.5	129	48.5
Industry	180	7.4	118	71.1	63	35.0	91	50.6
Construction	592	24.3	434	73.3	200	33.8	295	49.8
Services	1394	57.3	990	71.0	461	33.1	665	47.7
Origin country								
Ecuador	611	25.1	410	67.1	190	31.1	280	45.8
Morocco	625	25.7	485	77.6	307	49.1	351	56.2
Romania	601	24.7	428	71.2	164	27.3	289	48.1
Colombia	597	24.5	419	70.2	152	25.5	260	43.6
Living time in Spain								
< 2	295	12.1	212	71.9	80	27.1	135	45.7
2 to 6	1334	54.8	955	71.6	433	32.5	639	47.9
> 6	805	33.1	575	71.4	300	37.3	406	50.4
***Total***	***2434***	***100.0***	***1742***	***71.6***	***813***	***33.4***	***1180***	***48.5***

(n = 2434)

^a ^One person can perceive more than one type of discrimination.

^b ^Do not know/no answer: 0.5% missing value in the sample in age, 0.2% in educative level and 0.1% in economic activities.

**Table 3:** Distribution of the sample of immigrant workers in Spain and prevalence of the three categories of perceived discrimination for health outcomes

			Perceived discrimination^a^					
			
	Sample		Due to immigrant status		Due to physical appearance		Workplace related	
	
Health outcomes	n	%	n	Prevalence	n	Prevalence	n	Prevalence
Self-rated Health In origin country								
Good	2290	94.1	1632	71.3	753	32.9	1096	47.9
Poor	144	5.9	110	76.4	60	41.7	84	58.3
Self-rated Health In Spain								
Good	1998	82.1	1397	69.9	636	31.8	913	45.7
Poor	436	17.9	345	79.1	177	40.6	267	61.2
Change in self-rated health (origin- host country)								
No change/improved	2082	85.5	1460	70.1	670	32.2	959	46.1
Worse	352	14.5	282	80.1	143	40.6	221	62.8
Mental Health (In Spain) GHQ-12								
Good	1771	72.8	1185	66.9	518	29.2	731	41.3
Poor	662	27.2	557	84.1	295	44.6	449	67.8
Health problems self-perceived (Yes)^b^								
Muscular problems	642	26.4	512	79.8	251	39.1	371	57.8
Headache	812	33.4	624	76.8	293	36.1	460	56.7
Stress	806	33.1	638	79.2	300	37.2	439	54.5
Insomnia	375	15.4	308	82.1	165	44.0	234	62.4
Anxiety	423	17.4	348	82.3	170	40.2	252	59.6

(n = 2434)

^a ^One person can perceive more than one type of discrimination

^b ^Percentages are not mutually exclusive. Based on positive responses for each item.

Workers reporting discrimination were at a significantly higher risk of suffering all of the health problems analysed when compared with those not reporting discrimination (Table 4). These results were adjusted for all of the variables considered as possible confounders. Workers reporting workplace related discrimination were more likely to report self-perceived poor health (aOR 1.93; 95% CI 1.54-2.42), and more likely to report poor mental health (aOR 2.97; 95% CI 2.45- 3.60). Furthermore, the population reporting discrimination due to immigrant status was more likely to report anxiety (aOR 2.16; 95% CI 1.64- 2.83), and more likely to report insomnia (aOR 2.15; 95% CI 1.61- 2.86). The category of discrimination based on physical appearance demonstrated the weakest association with physical and mental health indicators (Table 4).

**Table 4:** Association between the three categories of perceived discrimination and poor health outcomes in immigrant workers in Spain

	Poor self-rated health		Muscular problems		Headache		Poor mental health GHQ = 12		Stress		Insomnia		Anxiety	
	
Perceived discrimination/health outcome	aOR	95% CI	aOR	95% CI	aOR	95% CI	aOR	95% CI	aOR	95% CI	aOR	95% CI	aOR	95% CI
No discrimination	1.00		1.00		1.00		1.00		1.00		1.00		1.00	
Discrimination:														
Due to immigrant status	1.75	1.35- 2.28	1.84	1.48- 2.29	1.52	1.25- 1.85	2.65	2.20- 3.35	1.92	1.57- 2.35	2.15	1.61- 2.86	2.16	1.64- 2.83
Due to physical appearance	1.43	1.14- 1.80	1.42	1.17- 1.72	1.21	1.01- 1.46	1.88	1.56- 2.26	1.33	1.10- 1.59	1.78	1.41- 2.24	1.46	1.17- 1.83
Workplace related	1.93	1.54- 2.42	1.70	1.41- 2.04	1.68	1.41- 2.00	2.97	2.45- 3.60	1.50	1.26- 1.79	2.06	1.64- 2.60	1.79	1.44- 2.23

Adjusted OR (95%CI)^a^. (n = 2434)

^a ^Adjusted OR (aOR) by sex, age, legal status, educative level, occupation, economic activities, country of origin, health status perceived before arriving to Spain and time in Spain.

Finally, Table 5 shows the calculations of population attributable proportions for reported worsened health of immigrants in Spain as compared to their health in the country of origin. Workplace related discrimination shows the strongest association with a decline in perceived health (aOR 2.20 95% CI 1.73-2.80). In addition, 40% of cases reporting worsening in self-perceived health were attributable to discrimination due to immigrant status, 37% of cases were attributable to perceived discrimination related to the workplace and finally 15% of cases were attributable to the perceived discrimination related to the physical appearance.

**Table 5:** Association between the three categories of perceived discrimination and population attributable proportion with change in self-rated health in immigrant workers in Spain

	Worse self-rated health in comparison with origin country			
	
Perceived discrimination	aOR^a^	95% CI	PAP (%)^b^	95% CI
No discrimination	1.00	1.00		
Discrimination:				
Due to immigrant status	1.93	1.45- 2.58	40.0	24.4- 53.1
Due to physical appearance	1.52	1.20- 1.94	14.8	6.3- 23.9
Workplace related	2.20	1.73- 2.80	36.8	26.1- 46.6

(n = 2,434)

^a ^Adjusted OR (aOR) by sex, age, legal status, educative level, occupation, economic activities, country of origin, health status perceived before arriving to Spain and time in Spain

^b ^Population attributable proportion: PAP

## Discussion

A high percentage of immigrant men and women in the study sample reported perceived discrimination, associated mainly with their condition as immigrants (after adjustment for potential sociodemographic and occupational confounding variables). However, sizeable segments of the population experienced discrimination due to physical appearance and related to the workplace. All three types of discrimination are associated with worse indicators of self-perceived health and with a decline in health status in Spain compared with health status in the country of origin.

The prevalence of perceived discrimination in our study is greater than that of the first survey on discrimination [33] carried out in a sample of immigrant populations in Spain in 2000 (characteristics of the participants were similar to those in the present study). The 2000 survey observed a perceived discrimination percentage of 19% in health institutions, 22% in public institutions (27.5% in current study) and 44% when looking for work or when at work (48.2% in current study). The values are also higher than in another study on discrimination carried out in 2002-2003 in minority ethnic groups in Spain [34] in which the discrimination reported by participants was 40% when looking for work or when at work, approximately 25% in public spaces (46% in current study), and 20% in institutions. These differences could be explained by the fact that the subjects in our sample have an increased time of residence in Spain.

A statistically significant association was found between discrimination type and the indicators of self-rated health. Scientific literature has shown that perceived experiences of discrimination have a negative effect on the health of people affected. In this sense, studies conducted in various countries have found associations between discrimination and mental health [35,36], physical health [37,38] and access to health-related services [39,40]. In Spain, two recent studies have observed that the perceived discrimination due to belonging to a certain social class, gender or ethnicity, among other causes, is associated with the affected population's poor physical and mental health [41,42].

The findings of this study are consistent with the existing literature on the subject [2-4,10]. The probability of reporting poor health was found to be similar to that of another study carried out with an immigrant population in the USA, with certain characteristics that were comparable with our study [43]. The association found regarding perceived discrimination and poor mental health coincides with similar studies on immigrant populations [15,44]. Furthermore, the perception of discrimination is related to the perception of specific health symptoms, such as stress [45], depression and anxiety [14,16], and sleep disorders [46]. The perception of this symptomatology in immigrants has been explained in the literature as the Ulysses syndrome or chronic stress syndrome [47], and it is expressed in men and women dealing with pain resulting from the migration process, for example, grieving the loss of contact with the family and the culture of the origin country, and the difficulties related to integrating at work and in society in general [48].

Everyday discrimination -which refers to general experiences of discrimination that occur on a routine basis- is correlated with health conditions, specifically in the workplace, after controlling for social factors [49]. In addition, certain types of discrimination may be more frequent in immigrants with more time in the host country, perhaps due to the accumulation of stressors or other factors related to social conditions [50,51]. This may be due to differences in specific working conditions of the collectives investigated: Immigrant workers express instability in contracts, difficulties in relationships at work and some characteristics of precarious work and employment [25]. In addition, differences in sampling strategies as well as inclusion/exclusion criteria of study participants and measures of perceived discrimination should be noted in comparison with previous studies [33,34,52]. For instance, the 2000 survey and the 2002-2003 study included other ethnic groups such as gypsy groups and those with African and Asian origins. Also, the 2000 survey included the perception of the Spanish-born toward different non-Spanish born groups in the country.

The existence of difficulties in accessing the job market, holding jobs of a low qualification despite meeting or exceeding the level of studies required [7,25,53], being subjected to conditions of job-related and social precariousness [23] and high temporality, or the absence of contracts for workers that do not have legal status [22] may also play an important role in perceived discrimination and subsequent health outcomes. Although there may be a process of selective migration of healthy people to host countries -sometimes referred to as the healthy immigrant effect-, the social conditions to which immigrants are subjected in the host country may be related to their decline in health compared with their state of health in their country of origin [25,54]. These factors could explain the temporality of the healthy immigrant effect as the immigrants spend more time in the host country (the health profile in immigrants with more time in Spain is similar to the native population in similar social classes) [55].

It should be made clear that one of the strengths of this study is that the methodology and information-gathering tools were carefully designed by means of previous qualitative research and the pilot study before the application of the questionnaire. This multi-method approach permitted improving the knowledge of characteristics related to the migration process and to the employment, work, and health conditions in the immigrant population with experience in the labour market in Spain.

However, in interpreting the results, it is important to take the study's limitations into account. Even though the study focused on important immigrant groups in Spain, the non-random sampling selection makes generalising conclusions about the population of immigrants in the country difficult. This is a common problem in investigating an immigrant population. This study obtained a response rate of 55.8% (similar to other research conducted on immigrant populations) due to difficulties in recruitment of participants and the fact that certain immigrants (for example, the undocumented) may be reluctant to take part in the study. The Spanish language requirement within the inclusion criteria for this study means that the sample may focus on a subset of immigrants who are already more acculturated than those who do not speak the language. For this reason it is important to study the associations indicated in this study in other immigrant groups living in Spain, especially those who do not speak Spanish. Another limitation of this study is the fact that it was carried out at the close of a robust economic cycle in Spain, which included important changes in the labour market. It is possible that this situation could result in differences in immigrants' perceptions of their general situation in an environment where many immigrants are increasingly excluded from the labour market [56].

Furthermore, the indicators studied were based on the interviewees' own perceptions of discrimination, and individual understanding of what is meant by "discrimination" is likely to vary depending on socio-demographic and other factors. For instance, in some cases, immigrants' responses to the question "Have you ever felt discriminated against?" present particular problems as we don't know whether they refer to other experiences of discrimination that occurred in their country of origin. Nevertheless it is important to clarify that most of the questions of the survey refer to the time spent in Spain and the primary or most recent job. In general, it is difficult to measure discrimination [57,58], and therefore research methods are based on the immigrants' own experiences in a specific context, as occurs in other studies [2-4,10].

## Conclusion

Despite the limitations, this study helps to characterize the relationship between discrimination, immigration and health, and identifies health indicators that could be useful for measuring the effect of self-perceived discrimination. Evidence from this study contributes to establishing causality, although a cohort study of foreign-born workers (as suggested) would provide stronger evidence of such causation. New hypotheses could emerge from other methodologies and further research would aid in establishing how certain associations are affected by contextual indicators regarding discrimination and health in Spain. Complementary analyses are currently being carried out focused on the impact of the economic crisis on immigrants in European countries and particularly in Spain, as economic changes have likely impacted the employment, working conditions and health of immigrants. Identification of the root causes of inequalities is necessary in order to act on their determining factors, perhaps through social policy mechanisms.

## Competing interests

The authors declare that they have no competing interests.

## Authors' contributions

All the authors participate in the ITSAL Project. All of them contribute with the data analysis, the written of the manuscript and the approbation of the final version to be submitted to the journal.

## Pre-publication history

The pre-publication history for this paper can be accessed here:

http://www.biomedcentral.com/1471-2458/11/652/prepub
